# Evaluating Detection and Diagnostic Decision Support Systems for Bioterrorism Response

**DOI:** 10.3201/eid1001.030243

**Published:** 2004-01

**Authors:** Dena M. Bravata, Vandana Sundaram, Kathryn M. McDonald, Wendy M. Smith, Herbert Szeto, Mark D. Schleinitz, Douglas K. Owens

**Affiliations:** * University of California San Francisco-Stanford Evidence-based Practice Center, Stanford, California, USA; † Stanford University School of Medicine, Stanford, California, USA; ‡ VA Palo Alto Healthcare System, Palo Alto, California, USA; § Kaiser Permanente, Redwood City, California, USA; ¶ Rhode Island Hospital, Providence, Rhode Island, USA; # Brown University School of Medicine, Providence, Rhode Island, USA

**Keywords:** bioterrorism, public health, expert systems, population surveillance, detection, diagnosis

## Abstract

We evaluated the usefulness of detection systems and diagnostic decision support systems for bioterrorism response. We performed a systematic review by searching relevant databases (e.g., MEDLINE) and Web sites for reports of detection systems and diagnostic decision support systems that could be used during bioterrorism responses. We reviewed over 24,000 citations and identified 55 detection systems and 23 diagnostic decision support systems. Only 35 systems have been evaluated: 4 reported both sensitivity and specificity, 13 were compared to a reference standard, and 31 were evaluated for their timeliness. Most evaluations of detection systems and some evaluations of diagnostic systems for bioterrorism responses are critically deficient. Because false-positive and false-negative rates are unknown for most systems, decision making on the basis of these systems is seriously compromised. We describe a framework for the design of future evaluations of such systems.

During the 2001 anthrax attacks, emergency response personnel, clinicians, laboratories, and public health officials were overwhelmed by requests for evaluation of suspicious powders and by calls from patients concerned about exposure to bioterrorism agents * (**1**–**4**)*. From October through December 2001, the New York City Bioterrorism Response Laboratory processed >3,200 environmental specimens *(2)*. In the 2 months after the discovery of anthrax in the Trenton, New Jersey, postal system, state police responded to >3,500 false alarms involving suspected anthrax *(3)*. These services were provided at great cost (e.g., as of November 2001, Philadelphia spent $10 million to investigate and test anthrax threats) *(3)*. Systems to detect bioterrorism agents in clinical and environmental samples and to diagnose bioterrorism-related illnesses are essential components of responses to both hoaxes and actual bioterrorism events.

First responders and public health officials require sensitive and specific detection systems that can identify bioterrorism agents early enough to take action that limits the spread of disease. Additionally, clinicians may benefit from diagnostic decision support systems, typically designed to generate a list of possible diagnoses for a given patient on the basis of clinical features, if these systems appropriately increase clinicians’ consideration of bioterrorism agents.

Under the auspices of the University of California-San Francisco-Stanford Evidence-based Practice Center, we prepared a comprehensive systematic review that evaluated the ability of available information technologies and decision support systems to serve the information needs of clinicians and public health officials during a bioterrorism response *(5)*. We describe the published evidence of evaluations of available detection systems and diagnostic decision support systems for bioterrorism-related illness. We then describe a framework that could be applied to future evaluations of these systems to determine whether they are likely to serve information needs of their users during a bioterrorism response.

## Methods

We performed a systematic review of descriptions and evaluations of systems for detection of bioterrorism agents and diagnostic decision support systems that could facilitate decision making for patients with undiagnosed bioterrorism-related illness. We provide a brief overview of our methods, which are described in detail elsewhere *(5)*.

We included reports of systems specifically designed to support the diagnosis of bioterrorism-relevant diseases or syndromes, as defined by the U.S. Department of Health and Human Services *(6)*. We also included reports of general diagnostic systems (e.g., systems that provide differential diagnoses based on a patient’s signs or symptoms), automated diagnostic test analysis systems, microbiologic test analysis systems for bioterrorism-specific agents, radiologic diagnostic systems that automatically make the diagnosis of pulmonary infiltrate or widened mediastinum, and rapid detection technologies. For all potentially relevant systems, reports were included if they, at a minimum, provided information about the system’s purpose, hardware requirements, type of information or sample required by the system, and type of information provided by the system.

### Literature Sources and Search Strategies

We searched five databases of peer-reviewed articles, (e.g., MEDLINE, National Technical Information Service, GrayLIT), Web sites of relevant government agencies (e.g., U.S. Department of Energy); and relevant nongovernmental Web sites. We included terms such as bioterrorism, biological warfare, decision support system, detection, diagnosis, radiology information systems, and public health. We also reviewed conference proceedings and reference lists of included articles.

### Study Selection and Data Abstraction

We screened peer-reviewed articles to determine if they met inclusion criteria. Two investigators blinded to study authors independently abstracted articles onto pretested abstraction forms. Data abstracted from each report varied, depending on the type of system described. For descriptions of detection systems, we abstracted information about the system’s portability, ability to run more than one sample at a time, and ability to detect more than one bioterrorism agent. For descriptions of diagnostic decision support systems, we recorded whether bioterrorism-related illnesses were included in the system’s knowledge base, how the system enabled updates of the probability of bioterrorism-related illness as an epidemic progresses, the method of reasoning used by the inference engine, and whether the system used a standard vocabulary.

### Criteria for Evaluating Reports of Included Systems

A complete description of the methods used to develop our evaluation criteria for reports of detection systems and diagnostic decision support systems can be found elsewhere *(5)*. Briefly, we reviewed reports of naturally occurring and bioterrorism-related outbreaks and solicited information from relevant experts to describe the detection and diagnostic decisions that clinicians and public health officials would have to make while responding to bioterrorism. We then described the capabilities of detection and diagnostic systems necessary to assist these decisions. We augmented this list of system characteristics with previously published standards for evaluating information technologies and diagnostic tests to develop evaluation criteria for systems designed to facilitate detection and diagnosis during a bioterrorism response *(5)*. We did not attempt to independently evaluate detection systems and diagnostic decision support systems; rather, we relied on information provided in the published reports about these systems.

## Results

We reviewed 17,510 citations of peer-reviewed articles, 6,981 Web sites of government agencies, and 1,107 nongovernmental Web sites. From these, we included 115 reports of 78 potentially relevant systems for a bioterrorism response (55 detection systems and 23 diagnostic decision support systems). We first present evaluative data about the detection systems and then the evaluative data about the diagnostic decision support systems.

### Detection Systems

We identified 55 detection systems including 4 systems that collect aerosol environmental samples; 14 particulate counters or biomass indicators that detect an increase in the number of particles in aerosol samples over baseline; 27 identification systems designed to rapidly detect bioterrorism agents collected from environmental, human, animal, or agricultural samples; and 10 systems that integrate the collection, identification, and communication of detection results *(5)*. Other detection systems exist; however, we describe all of the systems for which we found publicly available information through the search methods described.

Only 8 of the 55 detection systems had published evaluations (Table 1 and Table 2). No system was evaluated for all the evaluation criteria. Timeliness was described for 33 of the 55 detection systems. Of these systems, 20 were described in specific terms such as minutes or hours, whereas 13 systems were described in nonspecific terms such as “rapid” or “real-time”. Several reports included general statements about system sensitivity or detection limits; however, studies of only 1 of the 55 detection systems specifically reported both sensitivity and specificity (Table 2) * (**14**–**18**)*.

**Table 1:** Summary of evaluation data for detection systems and diagnostic decision support systems for a bioterrorism response

Evaluation criteria	Detection systems evaluated % (yes/total)	Diagnostic decision support systems evaluated % (yes/total)
Is the timeliness of diagnostic information described?	36 (20/55)	48 (11/23)
Are diagnostic sensitivity and specificity described?	1.8 (1/55)	13 (3/23)
Is the reference standard against which the system was compared described?	7 (4/55)	39 (9/23)
Are the system’s security measures described?	0	0
Is the evaluation of the system over a range of clinical situations or patient populations described?	0	0
Is the portability of the system described?	54 (15/28)	NA^a^
Is the system’s ability to run more than one sample at a time described?	10 (4/41)	NA
Is the system’s ability to detect more than one bioterrorism agent described?	32 (12/37)	NA
Is the system’s ability to detect either/both toxins and organisms described?	5 (2/37)	NA
Is the inclusion of all bioterrorism agents and associated illnesses in the system’s knowledge base described?	NA	26 (5/19)
Is the flexibility to update the probability of bioterrorism-related illness as the epidemic progresses described?	NA	0
Is the method of reasoning used by inference engine described?	NA	26 (5/19)
Is the use of standard vocabulary described?	NA	0

^a^NA; not applicable.

**Table 2:** Evaluation data for detection systems for bioterrorism agents^a^

System name	Purpose	Evaluation data^b^
Anthrax Sensor *(7)*	A portable detection system for “highly sensitive detection of biological agents within seconds” *(7)*.	Reported to be capable of detecting endotoxins at a level that is “20 times lower than previously achieved by similar devices” *(7)*.^c^
BioCapture *(8)*	A portable collection system for use by first responders.	Was compared to an All Glass Impinger (AGI) that collects samples into liquid and a Slit Sampler that impacts bacteria directly onto growth media and found to have a collection efficiency of 50%-80% relative to the AGI and 60%-125% relative to the Slit Sampler *(8)*.^c^
Digital Smell/Electronic Nose *(9)*	To detect and classify microorganisms according to the volatile gases given off during metabolism.	An array of 15 sensors was able to correctly classify 68 of 90 colonies containing 0 or 1 of 5 test organisms and an uninoculated control; however, it registered 22 of 90 as false-positives *(9)*.
Fluorescence-based array immuno-sensor *(10)*	To provide simultaneous, antibody-based detection of bioactive analytes in clinical fluids.	Bioterrorism agents intended to be detected include *Staphylococcus* enterotoxin B and F1 antigen from *Yersinia pestis*. It was unable to detect *S.* enterotoxin B levels (<125 ng/mL) in experimentally spiked urine, saliva, and blood products; sensitivity for F1 antigen from *Y. pestis* was reported at 25 ng/mL *(10)*.
LightCycler; Ruggedized Advanced Pathogen Identification Device (RAPID) *(11)*	LightCycler uses a PCR cycler for “real-time” quantification of DNA samples. RAPID is a rugged, portable system that uses LightCycler technology for field detection of bioterrorism agents.	RAPID is reported by the manufacturer to be **99.9% specific** (11). For each assay, the sensitivity is set to half the infective dose (for example, the infectious dose of foot and mouth disease is 10 virus particles; RAPID’s sensitivity is set to detect 5 virus particles [*11*]).^c^
MiniFlo *(12)*	For rapid, portable detection of multiple biological agents using flow cytometry.	Detected 87% of unknown biological agent simulants, including agents similar to anthrax and plague, with a false-positive rate of 0.4% *(12)*. Bioterrorism agents identifiable: *Y. pestis* and *Bacillus anthracis,* as well as other viruses, bacteria and proteins *(**12** ).*
Model 3312A Ultraviolet Aerodynamic Particle Sizer (UV-APS) and Fluorescence Aerodynamic Particle Sizer-2 (FLAPS-2) *(13)*	To detect living organisms in aerosols and nonvolatile liquids.	FLAPS-2 was able to detect 39 of 40 blind releases of stimulant aerosols (of particle ranging in size from 0.5 to 15 μm) at a distance of about 1 km with no false alarms during a 3-week period. In another trial, it was able to detect as few as 10 agent-containing particles per liter of air * (**13**,**4**)*.
Sensitive Membrane Antigen Rapid Test (SMART) and the Antibody-based Lateral Flow Economical Recognition Ticket (ALERT) * (**14**–**17**)*	A handheld antigen/antibody test for the rapid detection of bioterrorism agents.	When field tested during the Gulf War, the SMART system had an “alarmingly” high false-positive rate thought secondary to contamination (14). SMART tests are reported per the manufacturer to have a **96% to 99% sensitivity** and **94% to 99% specificity** for *Vibrio cholerae* O139 and O1) * (**14**–**17**)*

^a^PCR; polymerase chain reaction. ^b^Where possible, we report sensitivity and specificity data (and highlight them in bold); if the published reports did not provide these values directly but did provide sufficient data for them to be calculated, we performed these calculations. ^c^Denotes systems for which available evaluation data were from manufacturers’ Web sites only.

Of the four collection systems, we found evaluation data only for BioCapture (Meso Systems Technolgy, Inc., Albuquerque, NM), a device that has been used by fire departments in Seattle, Los Angeles, and New York among other sites to collect environmental samples for subsequent testing for bioterrorism agents *(8)*. Although its sensitivity and specificity were not described, the BioCapture system had a collection efficiency reported to be 50% to 125% relative to reference standards *(8)*. Reports on three other systems also included a comparison of the system under evaluation to a reference standard (Anthrax Sensor [*7*], MiniFlo [*12*], and the Fluorescence-based array [*10*]).

Most identification systems are limited in that they can evaluate a sample for only a single bioterrorism agent in each test cycle, they often run only a limited number of samples at a time, and they cannot test for many bioterrorism agents of concern (e.g., smallpox). None of the reports of the detection systems described methods for maintaining the security of the sample or test results or evaluated the systems in different clinical settings or among different populations. We found no studies that directly compared two or more systems in any given category.

In response to the 2001 anthrax cases, considerable interest was generated in the handheld antibody-based detection tests such as the Sensitive Membrane Antigen Rapid Test (SMART) (New Horizons Diagnostic Corp., Columbia, MD) and the Antibody-based Lateral Flow Economical Recognition Ticket (ALERT) * (**14**–**18**)*. Such systems use antibodies to recognize specific targets on the toxins, antigens, or cells of interest * (**13**,**14**)*. Limitations of these tests include nonspecific binding of the antibodies, which may lead to false-positive results and degradation of the antibodies over time, which may lead to false-negative results * (**13**,**14**)*. Additionally, these tests are limited by the availability of antibodies. Given concerns about the diagnostic sensitivity and specificity of hand-held, antibody-based tests when used during the anthrax attacks, the Federal Bureau of Investigation and Centers for Disease Control and Prevention performed an independent evaluation of these tests *(19)*. Although these results are not yet publicly available, the July 2002 Statement by the U.S. Department of Health and Human Services regarding hand-held assays for identification of *Bacillus anthracis* spores stated, “These studies confirm the low sensitivity of such assays and their potential to produce false-positive results with non-anthrax bacteria and chemicals. The performance of handheld assays for the detection of biological agents other than *B. anthracis* has not been evaluated and their use is also not recommended at this time” *(20)*. Instead, law enforcement should transport samples quickly to a Laboratory Response Network facility, where cultures will be performed and preliminary results made available within 12 to 24 hours *(20)*.

Several detection systems were designed in part, if not fully, by the military, and battlefield evaluations may have been performed. However, the paucity of publicly available information about such evaluations prevents conclusions about whether these systems will serve the detection needs of first responders and clinicians. Moreover, even if battlefield evaluation data were available, these systems would require additional study to confirm their utility for civilian users.

### Diagnostic Systems

We identified 23 diagnostic decision support systems that may enhance clinician consideration of bioterrorism-related illness. We found six general diagnostic systems, four systems designed to improve radiologic diagnoses, four telemedicine systems, and nine systems for other diagnostic purposes *(5)*. None has been formally evaluated with respect to a bioterrorism response; however, 15 diagnostic decision support systems had published evaluations for potentially analogous situations (Table 3).

**Table 3:** Evaluation data for diagnostic decision support systems for bioterrorism-related illness^a^

System name	Purpose	Evaluation data^b^
Clinical decision support system for detection and respiratory isolation of tuberculosis patients *(21)*	To automate the detection and respiratory isolation of patients with positive cultures and chest x-rays suspicious for TB.	In a retrospective analysis, the system increased the proportion of appropriate TB isolations in inpatients from 51% to 75% but falsely recommended isolation of 27 of 171 patients. In a prospective analysis, the system correctly identified 30 of 43 of patients with TB but not identify 21 of these patients (false-negatives). However, the decision support system identified 4 patients not identified by the clinicians *(21)*.
Columbia–Presbyterian Medical Center Natural Language Processor *(22)*	To automate the identification of 6 pulmonary diseases (including pneumonia) through analysis of radiology reports.	The system had a **sensitivity of 81%** (95% confidence interval [CI] 73% to 87%) and a **specificity of 98%** (95% CI 97% to 99%) compared to physicians who had an average sensitivity of 85% and specificity of 98% *(22)*.
Computer Program for Diagnosing and Teaching Geographic Medicine *(23)*	To provide a differential diagnosis of infectious diseases matched to 22 clinical parameters for a patient; also to provide general information about infectious diseases, anti-infective agents, and vaccines.	The computer program correctly identified 75% (222 of 295) of the actual cases and 64% (128 of 200) of the hypothetical cases of patients with infectious diseases *(23)*. The clinical diagnosis was included in the computer differential diagnosis list in 94.7% of cases. Among the cases included in this evaluation, several were for bioterrorism diseases *(23)*.
DERMIS * (**24**,**25**)*	To provide a differential diagnosis of skin lesions.	The system correctly diagnosed lesions 51% to 80% of the time and included the correct diagnosis among its top 3 choices 70% to 95% of the time (out of a total of 5,203 cases) * (**24**,**25**)*. The system was more accurate for dermatologist users than general practitioners.
Dxplain *(26)*	To provide a differential diagnosis based on clinician-entered signs and symptoms. The system includes descriptions and findings for potential bioterrorism agents, and is updated weekly to account for potential outbreaks.	In an evaluation of 103 consecutive internal medicine cases, Dxplain correctly identified the diagnosis in 73% of cases, with an average rank of 10.7 (the rank of a diagnosis refers to its position on the differential diagnosis—for example, the diagnosis with the greatest likelihood of being the actual disease is ranked first and the next most likely diagnosis is ranked second) *(26)*.
Fuzzy logic program to predict source of bacterial infection *(27)*	To use age, blood type, gender, and race to predict the cause of bacterial infections.	The program was able to correctly classify 27 of 32 patients into 1 of 4 groups based on demographic data alone *(27)*.
Global Infectious Disease and Epidemiology Network (GIDEON) *(28)*	To provide differential diagnoses for patients with diseases of infectious etiology. All potential bioterrorism agents as specified by CDC are included in the GIDEON knowledge base *(28)*.	Whereas medical house officers listed the correct diagnosis first in their admission note 87% of the time (for 75 of 86 patients), GIDEON provided the correct diagnosis for 33% (28 of 86 patients) *(28)*.
Iliad (and Medical HouseCall which is a system for consumers derived from Iliad) * (**29**–**31**)*	To provide a differential diagnosis based on clinician-entered signs and symptoms. The knowledge base is focused in internal medicine and was last updated in 1997.	In a multicenter evaluation, each of 33 users analyzed 9 diagnostically difficult cases. On average, Iliad included the correct diagnosis in its list of possible diagnoses for 4 of the 9 cases, and included the correct diagnosis within its top 6 diagnoses for 2 of the 9 cases. The differential diagnosis generated by Iliad is not dependent upon the level of training of the user * (**29**–**31**)*.
Neural Network for Diagnosing Tuberculosis *(32)*	To predict active pulmonary TB (using clinical and radiographic information) so that patients may be appropriately isolated at the time of admission.	The neural network correctly identified 11 of 11 patients with active TB (**100%** **sensitivity, 69%** **specificity)** compared with clinicians who correctly diagnosed 7 of 11 patients (64% sensitivity, 79% specificity) *(32)*.
PNEUMON-IA *(33)*	To diagnose community-acquired pneumonia from clinical, radiologic and laboratory data.	The decision support system correctly identified pneumonia in 4 of 10 cases, compared with between 3 and 6 cases for the clinician experts *(33)*.
Quick Medical Reference (QMR) *(34)*	To provide a differential diagnosis based on clinician-entered signs and symptoms.	One prospective study used QMR to assist in the management of 31 patients for which the anticipated diagnoses were known to exist in the QMR knowledge base. In the 20 cases for which a diagnosis was ultimately made, QMR included the correct diagnosis in its differential in 17 cases (85%) and listed the correct diagnosis as most likely in 12 cases (60%) *(34)*.
SymText *(**35**,**36**)*	To analyze radiology reports for specific clinical concepts such as identifying patients with pneumonia.	Average **sensitivity and specificity** for assessing the location and extension of pneumonia was 94% and 96% for physicians and **34% and 95%** for SymText. In selecting patients who are eligible for the pneumonia guideline, the area under the ROC curves was 89.7% for SymText and 93.3% for physicians *(**35**,**36**)*.
Texas Infectious Disease Diagnostic Decision Support System *(37)*	To provide a weighted differential diagnosis based on manually entered patient information.	The system was compared to a reference standard that missed the diagnosis of 98 of 342 cases of brucellosis. In 86 of the 98 patients, this system listed brucellosis in the top 5 diagnoses on the differential diagnosis list, and in 69 of these 98 patients, brucellosis was the only disease suggested by the system. The system missed the diagnosis in 12 of 98 patients. On average, without the system it took 17.9 days versus 4.5 days with the system to suspect the correct diagnosis *(37)*.
University of Chicago – Artificial Neural Network for Interstitial Lung Disease *(38)*	To help radiologists differentiate among 11 interstitial lung diseases by using clinical parameters and radiographic findings to develop a differential diagnosis.	Areas under the ROC curve obtained with and without the system output were 0.911 and 0.826 (p < 0.0001), respectively *(38)*.
University of Chicago – Computer Aided Diagnosis of Interstitial Lung Disease *(39)*	To aid in the detection of interstitial lung disease in digitized chest radiographs.	Areas under the ROC curve obtained with and without computer-aided diagnostic output were 0.970 and 0.948 (p *=* 0.0002), respectively *(39)*.

^a^TB, tuberculosis; CDC, Centers for Disease Control and Prevention; ROC, receiver-operating characteristic curve. ^b^Where possible, we report sensitivity and specificity data (and highlight them in bold); if the published reports did not provide these values directly but did provide sufficient data for them to be calculated, we performed these calculations.

The general diagnostic decision support systems are typically designed to assist clinicians develop a differential diagnosis list on the basis of patient-specific signs and symptoms. The included general decision support systems require manual entry of patient information by clinicians (*Table 2* and *Table 3*). They use probabilistic or rules-based inference to compare patient information with a knowledge base to generate a differential diagnosis list that is typically ranked in descending order of likelihood. Some of the systems provide additional information about the suspected diseases and suggest appropriate diagnostic tests if clinicians choose to pursue these diagnoses.

Three diagnostic decision support systems (Columbia–Presbyterian Medical Center Natural Language Processor, Neural Network for Diagnosing Tuberculosis, and SymText) were specifically evaluated for both sensitivity and specificity and typically performed better than physicians-in-training but not as well as experienced clinicians * (**22**,**32**,**35**,**36**)*. Also, the accuracy of the decision support systems decreased for difficult cases. The need for clinicians to manually enter patients’ data into diagnostic decision support systems, a laborious step that may be a barrier to the use of these systems and increases interuser variability, is eliminated by the few systems that automatically collect patient data from an electronic medical record * (**21**,**22**,**35**,**36**)*. For example, diagnostic decision support systems currently available in hospitals with electronic medical records provide clinicians with an estimate of the likelihood of community-acquired pneumonia or active pulmonary tuberculosis based exclusively on data collected from the medical record * (**21**,**32**,**33**,**35**,**36**)*.

Two infectious disease diagnostic decision support systems, The Computer Program for Diagnosing and Teaching Geographic Medicine and GIDEON, included most of the bioterrorism-related organisms in their knowledge bases * (**23**,**28**)*. In an evaluation of The Computer Program for Diagnosing and Teaching Geographic Medicine, the system correctly identified 222 (75%) of 295 cases and 128 (64%) of 200 hypothetical cases *(23)*. The clinical diagnosis was included in the computer differential diagnosis list in 95% of cases. Several cases included in this evaluation were for the causative agents of anthrax, brucellosis, cholera, Lassa fever, plague, Q fever, and tularemia.

An evaluation of GIDEON compared its diagnostic accuracy to that of medical house officers admitting 86 febrile adults to the hospital *(28)*. The house officers listed the correct diagnosis first in their admission note 87% (75/86) of the time compared with 33% (28/86) for GIDEON (28). To limit the differential diagnosis provided by the system, users enter the geographic area where the outbreak occurred. This geographic information is compared with the known areas of natural occurrence. Adding this geographic information could falsely decrease the probability of disease if a bioterrorism agent were used in a region that had little naturally occurring disease from that organism.

Many diagnostic decision support systems use probabilistic information about the likelihood of disease. Because bioterrorism-related illness is relatively rare, in the event of bioterrorism these systems will have inappropriately low pretest probabilities for bioterrorism agents. Only Dxplain was described as being able to change the probability of disease based on information about suspected bioterrorism events to improve the system’s performance *(26)*. Additionally, no report specifically described restricting access to the system by user type or other security measures.

## Discussion

We systematically examined the 115 published reports of 55 detection and 23 diagnostic systems for bioterrorism responses. We found that technologies are increasingly available to assist detection and diagnostic tasks involved in a bioterrorism response but that only 23 systems were evaluated according to one or more evaluation criteria. Of these, 13 were compared to a reference standard test, none was evaluated in a range of clinical situations or in different populations, and only 4 reported both sensitivity and specificity. This remarkable lack of published evaluation data markedly affects both purchasers and users of such technologies. Decision makers will find it difficult to choose systems for purchase as they make resource allocations for bioterrorism preparedness. Users of these technologies may find it difficult to interpret the detection and diagnostic information provided by these systems. For example, if a first responder were asked to determine the presence or absence of a bioterrorism agent in a suspicious powder using a detection system with a high false-positive rate, he may cause unnecessary evacuation of environments suspected to be contaminated, work stoppages, and anxiety. In contrast, if a first responder used a system with high false-negative rate, he may have missed a bioterrorism agent, thereby risking excessive disease and death. Thus, for detecting and diagnosing bioterrorism-related illness, users require systems that are both highly sensitive and specific. Because ideal systems with near perfect sensitivity and specificity do not currently exist, and may be very difficult to produce for use in the field, users of available systems are faced with substantial challenges when interpreting the results from diagnostic tests.

**Figure 1:**
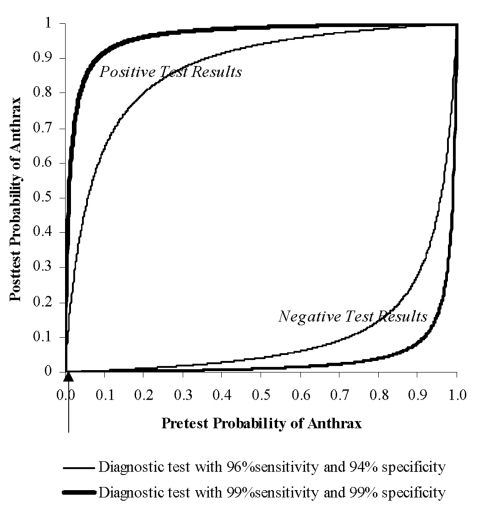
Effect of sensitivity, specificity, and pretest probability on posttest probability of anthrax’s being present. Upper curves show the posttest probability of anthrax’s being present after a positive detection or diagnostic test result. Lower curves show the posttest probability of anthrax’s being present after a negative detection or diagnostic test result. Separate curves are drawn for two diagnostic tests described in the text: one with 99% sensitivity and 99% specificity (thick) and another with 96% sensitivity and 94% specificity (thin). The arrow marks a pretest probability of disease of 0.0014, which relates to the example described in the text.

We can illustrate the critical importance of sensitivity and specificity of detection systems by considering the anthrax attacks of fall 2001. The Trenton, New Jersey, state police evaluated >3,500 samples of suspicious powders, and none contained anthrax *(3)*. For the purpose of illustration, let us assume that, before testing, 5 of these 3,500 samples were estimated to contain anthrax (i.e., pretest probability equals 0.0014). If a detection test had a sensitivity of 96% and specificity of 94% (i.e., the lower range reported for SMART/ALERT), we can calculate the posttest probability of anthrax with both positive and negative test results by using Bayes’ theorem *(40)*. If such a detection system indicated a positive result, the probability that the sample contained anthrax would be approximately 2%. That is, 98% of the positive results would be false-positives. If the system indicated a negative result, the probability of anthrax in the sample would be 0.006%. Thus, the test would be useful when negative, but provide little help if positive. If the sensitivity and specificity of the detection systems were both 99% (i.e., the upper range reported for SMART/ALERT), the posttest probability after a positive test would be 12%, and after a negative test, virtually 0. Thus, even with a specificity of 99%, only 12% of samples indicated as positive would contain anthrax, and 88% would be false-positive results. This relationship between a diagnostic test’s sensitivity and specificity and the pretest probability of disease is depicted in Figure 1.

This example illustrates the challenges for bioterrorism detection systems. Testing will often be done at very low pretest probabilities. Thus, a bioterrorism detection system must have very high specificity or the vast majority of positive results will be false-positives. In contrast, under circumstances when testing is performed at relatively high pretest probability (for example, in a heavily contaminated building), a negative test result will only be convincing if the sensitivity of the system is very high. Thus, interpretation of diagnostic test results requires ongoing evaluation of the pretest probability of a bioterrorist attack.

A common approach to minimize false-negative and false-positive results is to perform confirmatory tests after initial tests are completed. Such use of tests in sequence creates additional difficulties interpreting their results. Under ideal conditions for sequential tests, we can use the posttest probability of the first test as the pretest probability of the second test to calculate the posttest probability after the confirmatory test. This calculation is only accurate, however, if the sensitivity and specificity of the confirmatory test are the same regardless of whether the initial test was positive or negative. If this circumstance is not met, investigators must measure the sensitivity and specificity of the confirmatory test in samples or populations with negative and positive results on the initial test. This information is rarely available.

Sensitivity and specificity are defined only for a test with two outcomes, such as positive or negative. For tests with multiple outcomes, such as a detection system that identifies multiple agents, investigators can characterize the performance of the test with likelihood ratios *(40)*. Users can calculate the posttest probability for such a test with the likelihood ratio form of Bayes’ theorem *(40)*.

Evaluation of diagnostic decision support systems is more complex because the purpose of these systems is typically to generate a differential diagnosis. Thus, the evaluation determines the appropriateness of the differential diagnosis, and perhaps, if the diseases in the differential diagnosis are ranked, how high the correct disease is ranked. Specific recommendations for evaluation of decision support systems have been published elsewhere *(5)*. The studies of the diagnostic decision support systems included in Table 3 use a variety of approaches to assess the performance of the systems. However, only two have been evaluated specifically for capture of diseases caused by bioterrorism agents in the differential diagnosis list. Many of the systems require manual entry of patient data, and none are in widespread use. Based on the available evidence, we conclude that the available diagnostic decision support systems will be of limited usefulness in response to a bioterrorism event.

### Recommendations for Study Design of Detection Systems

For the purpose of evaluation, detection systems have much in common with diagnostic tests. Published guidelines for evaluating diagnostic tests are well established and promote study designs that provide unbiased estimates of both sensitivity and specificity (or likelihood ratios) relative to an acceptable reference standard, in the appropriate clinical population or setting.

**Figure 2:**
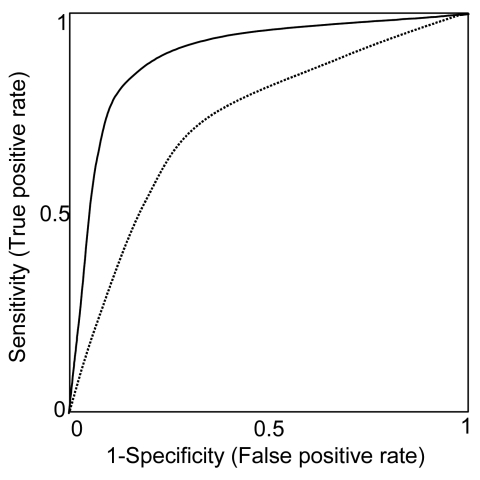
Receiver-operating characteristic curves (ROC). Each point along a ROC represents the trade-off in sensitivity and specificity, depending on the threshold for an abnormal test. Here, two hypothetical diagnostic tests are compared. The diagnostic test represented by the unbroken ROC curve is a better test than that represented by the broken ROC curve, as demonstrated by its greater sensitivity for any given specificity (and thus, greater area under the curve).

The first important design consideration is that both sensitivity and specificity (or likelihood ratios) must be measured relative to an appropriate reference standard. Many of the studies included in our review measured only sensitivity or specificity. Because sensitivity and specificity are jointly determined by the choice of threshold for a positive (or abnormal) test, either sensitivity or specificity can be made arbitrarily high at the expense of the other. Thus, reporting one without the other is not informative. Reporting both sensitivity and specificity for a variety of thresholds for abnormal tests as a receiver operating characteristic (ROC) curve (Figure 2) is preferable. ROC curves are useful because differences in sensitivity and specificity of two tests could be due either to real differences in the accuracy of the test or to the use of a different threshold for an abnormal test. When results are reported as an ROC curve, no such confounding will occur.

To develop unbiased estimates of sensitivity and specificity, studies of detection systems should use an appropriate reference standard test, the reference standard should be applied to all samples, the tests should be interpreted while blinded to results of the reference standard, and the samples or patient population should resemble as closely as possible the populations in which the system will be used *(40)*. The reference standard should be used for all positive and negative samples. Selective use of the reference standard, for example, using the reference standard only on samples that are positive on the test under consideration, creates so-called test referral bias which can produce overestimates of sensitivity and underestimates of specificity *(40)*. Test-interpretation bias may occur if the result of the detection system is not determined while blinded to the reference test (and vice versa). This bias causes an artificial concordance between the detection system and reference test, which results in overestimates of both sensitivity and specificity. Finally, the detection system should be evaluated under the most realistic conditions possible, which may be difficult to implement for bioterrorism agents given the range of conditions from hoaxes with no cases to real situations with a number of cases.

Evaluations of detection systems are ongoing *(19)*. We expect with the heightened attention to bioterrorism preparedness planning that the systems for both detection and diagnosis will improve, as will their evaluations. Evaluations that adhere to the principles for design of studies of diagnostic tests will provide substantially more information than is now available and will help users interpret the results provided by these systems. Our review of 78 detection and diagnostic systems found that many of the evaluations performed to date are critically deficient. Further evaluative studies will delineate the usefulness of these systems.
